# Internalized Gold Nanoparticles Do Not Affect the Osteogenesis and Apoptosis of MG63 Osteoblast-Like Cells: A Quantitative, In Vitro Study

**DOI:** 10.1371/journal.pone.0076545

**Published:** 2013-10-02

**Authors:** Shiao-Wen Tsai, Jiunn-Woei Liaw, Ya-Chen Kao, Meng-Yu Huang, Chia-Ying Lee, Lih-Rou Rau, Chiung-Yin Huang, Kuo-Chen Wei, Tzu-Chen Ye

**Affiliations:** 1 Graduate Institute of Biochemical and Biomedical Engineering, Chang Gung University, Taoyuan, Taiwan; 2 Center for Biomedical Engineering, Chang Gung University, Taoyuan, Taiwan; 3 Department of Mechanical Engineering, Chang Gung University, Taoyuan, Taiwan; 4 Department of Neurosurgery, Chang Gung Memorial Hospital, Linkou, Taiwan; 5 College of Medicine, Chang Gung University, Taoyuan, Taiwan; 6 Department of Medical Imaging and Radiological Sciences, Chang Gung University, Taoyuan, Taiwan; 7 Department of Nuclear Medicine, Chang Gung Memorial Hospital, Linkou, Taiwan; 8 Molecular Imaging Center, Chang Gung Memorial Hospital, Linkou, Taiwan; University of Notre Dame, United States of America

## Abstract

The long-term toxicity effects of gold nanoparticles (GNPs) on the proliferation and differentiation of a progenitor cell line, MG63 osteoblast-like cells, was investigated. These cells were treated for 20 hours with two media that contained 10 nm GNPs at concentrations of 1 ppm and 10 ppm. The mitosis of the GNP-treated MG63 was observed after at least 21 hours using dark-field and fluorescence microscopy. The TEM, LSCM and dark-field hyperspectral images indicated that the late endosomes in cells that contained aggregated GNPs were caused by vesicle fusion. Subsequently, after 21 days of being cultured in fresh medium, the specific nodule-like phenotypes and bone-associated gene expression of the treated MG63 cells exhibited the same behaviors as those of the control group. Statistically, after 21 days, the viability of the treated cells was identical to that of the untreated ones. During the cell death program analysis, the apoptosis and necrosis percentages of cells treated for 8 or fewer days were also observed to exhibit no significant difference with those of the untreated cells. In summary, our experiments show that the long-term toxicity of GNPs on the osteogenetic differentiation of MG63 is low. In addition, because of their low toxicity and non-biodegradability, GNPs can potentially be used as biomarkers for the long-term optical observation of the differentiation of progenitor or stem cells based on their plasmonic light-scattering properties.

## Introduction

Molecular imaging is a potential method for detecting and imaging specific cells and molecules to understand their particular interactions *in vivo*. This method has proven useful in proteomics and genomics research as well as for the diagnosis and therapy of certain diseases. Over the past few decades, numerous dye molecules with different excitation and emission spectra, including those conjugated with nanoparticles, have been developed as nanoprobes for labeling and tracking specific cells. However, because of photobleaching of the dye molecules, fluorophores are not suitable for long-term observational studies. Instead, quantum dots (QDs) and metallic nanoparticles (MNPs) have been developed as molecular probes for long-term studies. The advantages offered by QDs include a narrow emission spectrum, a high level of expression, and long-term use. However, some previous studies have demonstrated that QDs impair the differentiation of human bone mesenchymal stem cells [1]. As a consequence, great effort has been devoted to the development of high-sensitivity and high-resolution optical nanoprobes with low detection limits. Among these new nanoprobes, MNPs, including gold nanoparticles (GNPs), gold nanorods and magnetic nanoparticles are promising biomarkers that have recently been developed. Recently, GNPs have attracted substantial interest due to their unique surface plasmon resonance (SPR) property [2]. The SPR band of GNPs is strongly dependent on the size and shape of the nanoparticles. In general, the SPR band is red-shifted as the size or aspect ratio increases. Because of the tunable SPR of GNPs, they have been investigated extensively for biomedical applications, including biosensing [3], molecular imaging [4-7] and photothermal therapy [8,9].

To date, numerous studies on how to control the size, shape and surface modifications of GNPs have been conducted to uncover more versatile applications for the particles. For example, exploitation of different affinities of single- and double-stranded oligonucleotides adsorbed on the surface of GNPs may allow the design of a visual colorimetric hybridization assay to detect single-base mismatches between a probe and a target [10]. The application of GNPs immobilized with hyaluronic acid as nanoprobes for the detection of reactive oxygen species (ROS) and hyaluronidase has been proposed [11]. GNPs conjugated with epidermal growth factor receptor antibody (anti-EGFR) [12] or with folic acid [13] have been developed to increase their specific uptake efficacy by cells. The size and surface properties of GNPs also affect the biodistribution of nanoparticles throughout the body. A few reports have indicated that GNPs that persist in the body for more than 2 months could induce gene expression in large organs [14]. Thus, before GNPs are utilized as diagnostic and therapeutic agents, their long-term safety must be evaluated.

Recently, a number of researchers addressed the cytotoxicity of GNPs, and their results are summarized in Table 1. However, no consensus has thus far been reached. Some researchers have shown that cytotoxicity may result from the effects of shape, size [15-18], surface modifications [19-22], or the dosage of GNPs [23,24]. However, others have concluded that GNPs exhibit no significant cytotoxicity [25,26]. We attribute this contradiction to differences in the conditions used in these previous studies, including the size, surface modification characteristics, and uptake dosages of GNPs. Therefore, in this study, we prepared GNPs with a diameter of 10 nm with no specific functionalization and quantified the concentration of GNPs in the medium and the uptake dosage per cell using inductively coupled plasma-optical emission spectrometry (ICP-OES). Our purpose was to investigate the long-term effects of GNPs on the apoptosis, proliferation and differentiation of MG63 osteoblast-like cells.

**Table 1:** Studies of the cytotoxicity of GNPs.

**Reference**	**Size of GNPs**	**Surface modification of GNPs**	**Concentration of GNPs**	**Type of cell-line examined**	**Effect of cytotoxicity**
Arnida et al. [20]	30, 50 and 90 nm	PEG-coated	1.5 nM	Human prostate carcinoma PC-3 cells	no harmful effect
Chen et al. [44]	3, 5, 50 and 100 nm	NA	8 mg/kg/week	BALB/C mice	no harmful effect
	8, 12, 17 and 37 nm				induced severe sickness
Cho et al. [23]	17.7 nm	positive surface by poly(allyamine hydrochloride)	0.027 nM	Human breast cancer cells (SK-BR-3)	non-cytotoxic
		negative surface by citrate			
		neutral surface by poly(vinyl alcohol)			
Cho et al. [37]	13 nm	PEG-coated	0.17 to 4.26 mg/kg	BALB/C mice	Induce acute inflammation and apoptosis
Connor et al. [36]	18 nm	GNPs with a variety of surface modifiers such as citrate, biotin and CTAB	25µM	K562 leukemia cell line	not inherently toxic
Goodman et al. [38]	2 nm	positive surface by ammonium-functionalized	0.38-, 0.75-, 1.5- and 3 µM	Cos-1, red blood cells and bacterial (*Escherichia coli*)	moderate
		negative surface by carboxylate-substituted			non-cytotoxic
Male et al. [25]	5-6 nm	fluorescent gold nanoparticles	up to45μM	Chinese hamster lung ﬁbroblast V79 cells	non-cytotoxic
Mironava et al.[23]	13 nm	NA	95, 142 and 190 µg/ml	Primary human dermal fibroblasts CF-31 cells	GNPs are toxic for human dermal fibroblasts
	45 nm		13, 20 and 26 µg/ml		
Paino IMM et al.[43]	7-20 nm	citrate or PAMAM	1.0 and 50.0 µM	HepG2 and human peripheral blood mononuclear cell (PBMC)	both types of GNPs exhibit in vitro genotoxicity and cytotoxicity
Pan et al.[15]	0.8 to 15 nm	Triphenylphosphine monosulfonate and tris-sulfonated triphenylphosphine	1.4 nm with IC50 from 30 to 46 µM; 0.8 nm with 250 µM; 1.2 nm with 140 µM and 1.8 nm with 230 µM	SK-Mel-28 human melanoma, HeLa human cervix carcinoma, L929 mouse fibroblasts, and J774A1 mouse macrophages	1.4 nm were highly toxic and both smaller gold and larger gold were nontoxic
Pernodet et al. [21]	13.1±1.4 nm	NA	0 to 0.8 mg mL^-1^	Human dermal fibroblasts (CF-31)	damaged internal cell activities
Shukla et al. [39]	3 to 8 nm	Reduced gold, and lysine-, or poly-L-lysine-substituted	10-, 25-, 50-, and 100 µM	RAW264.7 macrophage cells	non-cytotoxic
Soenen et al. [24]	4 nm	poly(methacrylic acid)	10, 20, 50, 100, and 200 nM,	neural progenitor cells (C17.2), rat pheochromocytoma cells (PC12) and Primary human umbilical vein endothelial cells	a cytotoxic effect was observed higher than 50 nM, but not cytoxicis at 10 nM
Tarantola et al. [19]	43±4 nm	CTAB	9 µg/ml	epithelial cell	spherical GNPs are more toxic than rod-like GNPs.
Taylor et al.[41]	15 nm	NA	50 µM	bovine endothelial cell line cells (GM7373)	a cytotoxic effect was observed at 50 µM and above, but not cytoxicis at 25 µM or below
Thakor et al. [24]	60 nm	silica shell,	1–100 nanoparticles per cell	Pathogen-free HeLa and human hepatocellular carcinoma (HepG2) cell	no cytotoxicity
Yen et al.[40]	2.8-, 5.5-, and 38 nm	NA	1-, and 10 ppm	J774 A1 murine macrophages	1 ppm showed no cytotoxicity; 10 ppm showed significant cytotoxicity
Yi et al. [42]	20 nm	NA	0.1, 0.2 and 1 nM	mesenchymal stem cells	promoted the osteogenic differentiation and inhibited the adipogenic differentiation

## Materials and Methods

### Synthesis of GNPs

In our experiments, GNPs were synthesized via the citrate reduction of a gold salt [27]. Briefly, 100 ml of deionized water containing 10 µl of 0.5 M HAuCl_4_ and 3 ml of 1% sodium citrate was heated to boiling. The sizes of the GNPs were controlled by tuning the time that the solution was maintained at boiling temperature. The other chemicals were of reagent grade and were purchased from Sigma-Aldrich. The sizes of the nanoparticles were measured by randomly choosing 100 GNPs from TEM (JEOL 1230) images. The surface potential was measured with a Zetasizer (Nano-ZS90 system, Malvern). The concentrations of GNPs in the media were measured using ICP-OES.

### Cell Culture

In this research, MG63 osteoblast-like cells (human osteogenic sarcoma, BCRC number: 60279) were used to investigate the nanotoxicity of GNPs on cellular behaviors. The MG63 cells represent an immature osteoblast phenotype that can be induced to differentiation [28-30]. Therefore, the MG63 cells should secrete bone matrix constituents for bone matrix mineralization under suitable induction culture conditions. Moreover, MG63 cells display rapid cell growth without contact inhibition, so the cells could display an aggregation phenotype after long-term culture times [31]. Dulbecco’s Modified Eagle’s Medium containing 10% fetal bovine serum and 1% antimicrobial agent was used. The induction medium was supplemented with 50 µg ml^-1^ ascorbic acid and 10 mM glycerol 2-phosphate disodium salt hydrate. The cells were incubated at 37 °C in a humidified atmosphere of 5% CO_2_, and the culture medium was changed once every three days.

### Cell Growth Assay

The effects of GNPs on the cell growth and doubling time were analyzed using a trypan-blue exclusion method. The MG63 osteoblast-like cells were pre-cultured in 24-well tissue culture plates (5 × 10^4^ cells well^-1^) for 4 hours, after which fresh medium supplemented with GNPs at a concentration of 1 ppm or 10 ppm was added to the wells. A normal culture was used as the control group. The culture medium was changed once per three days. The cells were lifted off the culture plate using trypsin and then treated and washed; the cells were subsequently stained with trypan blue and counted with a hemocytometer. The effect of the GNP concentration on the viability of the cells was measurement in triplicate, and the experiments were repeated three times. The total numbers of cells were counted on days 1, 3, 7, 14 and 21.

### Cell Optical Imaging

Cellular morphology was investigated by examining the staining of two cell components: the F-actin cytoskeleton, which was fluorescently stained with Texas Red-X phalloidin, and the nucleus, which was stained with Hoechst 33258. After being cultured for 1, 7, 14 and 21 days, the cells were fixed in a 4% para-formaldehyde solution for 15 min at room temperature and then treated with 0.1% Triton X-100 for 5 min. To reduce non-specific background staining, the samples were blocked with 1% bovine serum albumin (BSA) in PBS for 30 min. After the BSA solution was aspirated, the samples were incubated with 200 µl of Texas Red-X phalloidin for 20 min. Then, to stain the DNA in the nuclei of these cells, the samples were incubated with Hoechst 33258 solution for 15 min. After three 5 min washes with PBS, the samples were observed under a laser scanning confocal microscope (LSCM, Zeiss LSM 510 META). The light scattered from GNPs is monochromatic, and its wavelength is the same as that of the CW laser. In this study, a diode-pumped solid-state laser with a wavelength of 561 nm was used to obtain images of GNPs. In addition to using the LSCM to observe the cell morphology and distribution of GNPs inside the cells, we also utilized a dark-field hyperspectral imaging system to verify the optical properties of the intracellular particles. The dark-field hyperspectral imaging system (Cytoviva) included an Olympus BX51 microscope (Tokyo, Japan) and a high numerical dark-field condenser (U-DCW, 1.2-1.4, Cytoviva). A 100_oil Iris objective was used and the dark-field and bright-field images were captured with an Olympus DP72 single-chip color CCD camera (Tokyo, Japan).

### Quantifying Intracellular GNP Dosages

The MG63 osteoblast-like cells were pre-cultured in 24-well tissue culture plates (5 × 10^4^ cells well^-1^) for three hours. After the preculture, fresh medium, supplemented with 1 ppm or 10 ppm GNPs, was added to the wells. The cultured MG63 cells were harvested at 24, 48 and 72 hours, and the cells were subsequently washed three times with PBS. Then, 200 µl of aqua regia was added to the cultured plate to dissolve the intracellular GNPs. The GNP dosages per cell were measured via ICP-OES. Each sample was measured in triplicate, and the experiments were repeated three times.

### Gene Expression Analysis Using Real-time PCR

After 7, 14, or 21 days of culturing, the total RNA was extracted from each sample with TriReagent according to the manufacturer’s instructions. The concentration and purity of each sample were assessed from the absorbance at 260 nm and the ratio between the absorbances at 260 nm and 280 nm, respectively. Real-time PCR was used to determine the levels of OPN, OCN, collagen type I, and 18S ribosomal RNA. Table 2 lists the sequences of the oligonucleotides that were used as PCR primers. Briefly, the reaction volume (25 µL) included 12.5 µL of SYBR Green PCR Master Mix (Protech SA-SQGLR-V2), 3 µL of diluted cDNA (15 ng), 0.5 µL of MgCl_2_ (25 mM), 4 µL of ddH_2_O and 2.5 µL each of the forward and reverse primers (10 µM). After being initially denatured at 94 °C for 15 min, the target genes were amplified with 40 cycles of denaturation at 94 °C for 15 s and annealing at 62.5 °C for 60 s. Real-time PCR reactions were performed with an iQ5 Gradient Real Time PCR system (Bio-Rad). The levels of RNA expression were determined according to the 2^–∆∆Ct^ method. The expression levels of the target genes were calculated by normalizing the mRNA level of a particular gene against that of the 18S ribosomal RNA as an internal control. The fold changes were calculated using the following formulas:

$$\begin{array}{c} Sample\;\Delta C_{t}=C_{t\;sample}\;-C_{t\;18S\;ribosomal}\\ \Delta \Delta C_{t}\;=Sample\;\Delta C_{t}\;-Control\;\Delta C_{t}\\ Fold-change\;of\;the\;sample\;vs.\;control\;=2^{-\Delta \Delta C_{t}} \end{array}$$

**Table 2:** Oligonucleotide primer for PCR amplification.

**Gene**	**Primer sequence: sense/antisense**
Collagen Type I	5’-CGGAGGAGAGTCAGGAAG-3’
	5’-CAGCAACACAGTTACACAAG-3’
Osteopontin	5’-AAGCGAGGAGTTGAATGG-3’
	5’-CTCATTGCTCTCATCATTGG-3’
Osteocalcin	5’-CAGCGAGGTAGTGAAGAGAC-3’
	5’-GCCAACTCGTCACAGTCC-3’
18S ribosomal RNA	5’-GAAGATATGCTCATGTGGTGTTG-3’
	5’-GTCTTAGGTGCGGTCATGTTC-3’

### Cell Death Program Analysis

To examine the effect of GNPs on the cell death program, Annexin V/PI double-labeling and FACS analysis were used for the detection of phosphatidylserine externalization due to phosphatidylserine translocation from the inner surface to the outer surface of the plasma membrane; the appearance of phosphatidylserine is one of the indicators of the early apoptosis of cells [32]. Briefly, the cells were pre-cultured in the medium for three hours and then treated either with or without 10 ppm GNPs for an additional 20 hours. After the medium that contained GNPs was aspirated, the cells were incubated in fresh medium in preparation for a cell death program analysis every two days for a period of eight days. At the end of the incubation period, the cells were harvested and mixed with 100 µl of annexin-binding buffer (10 mM HEPES: NaOH, pH 7.4, 140 mM NaCl, 2.5 mM CaCl_2_), 5 µl of Annexin V–FITC and 1 µl of PI. The cell mixture was kept at room temperature in the dark for 15 min. After the cell mixture was washed three times in PBS, the cells were resuspended in binding buffer (400 µl) prior to analysis using a FACS Calibur flow cytometer (Becton Dickinson). Each sample was analyzed, and the fluorescence intensity was measured on a four-decade log scale. Cells treated with H_2_O_2_ were used as a positive control. The percentage of cell death was quantified using the Cell Quest software package.

### TEM Imaging of GNPs

To understand the distribution and location of nanoparticles in the cells, the cells were seeded in 6-well culturing plates (5 × 10^4^ cells well^-1^) and grown to confluence. The cells were then treated with the GNPs at a concentration of either 1 ppm or 10 ppm for an additional 24, 48 or 72 h. After the treatment, the cells were detached and centrifuged, and the obtained cell pellet was washed with PBS. Subsequently, the pellet was fixed with 2.5% glutaraldehyde in PBS for 30 min and then postfixed in 1% osmium tetroxide in PBS for 1 hour. After dehydration, ultrathin, spur-embedded sections of the pellet were stained with uranyl acetate and lead citrate. TEM images of GNPs were subsequently obtained.

### Statistical Analyses

Experiments were conducted in triplicate, and the results are expressed as the means ± SDs. Statistical analyses were performed using the SPSS v.10 software package. Particles sizes were analyzed using Student’s t-test. Cellular viability, alkaline phosphatase activity and gene expression were analyzed by nonparametric Kruskal-Wallis H-test, if significant at a value of *p*<0.05, and then individual Mann-Whitney U-test was conducted for differences among groups. Differences of *p*< 0.05 were considered statistically significant.

## Results

Previous studies have indicated that smaller particles or higher dosages more readily induce cell damage, as summarized in Table 1. Accordingly, we prepared two culture media that contained GNPs at concentrations of 1 ppm and 10 ppm to study the long-term toxicity of GNPs on the osteogenetic differentiation of MG63. The average size of the GNPs was 10.76 ± 1.4 nm, and the GNPs exhibited a negative surface potential of 42.1 ± 0.65 mV. The energy-dispersive (EDS) spectrum of the GNPs shows that their surfaces do not contain a considerable number of oxygen atoms, which demonstrates that the GNPs utilized in the present study did not contain any specific functional groups even though they were synthesized via the citrate reduction of a gold salt (File S1). Because the GNPs had a negative surface potential, the GNPs utilized in this work are also called citrate-capped GNPs, a name that is sometime used in others reports. Because the doubling time of the MG63 cells is approximately 24 hours, we treated MG63 with the two media for less than 24 hours to observe the effects of the concentration of GNPs on cell attachment and phenotype. According to our experimental results, the doubling time of MG63 was not affected by the GNPs at concentrations of 1 ppm and 10 ppm (File S2). Control cells incubated in fresh medium were also prepared for reference.

### Effect of GNPs on Cell Division, Attachment and Phenotype

**Figure 1:**
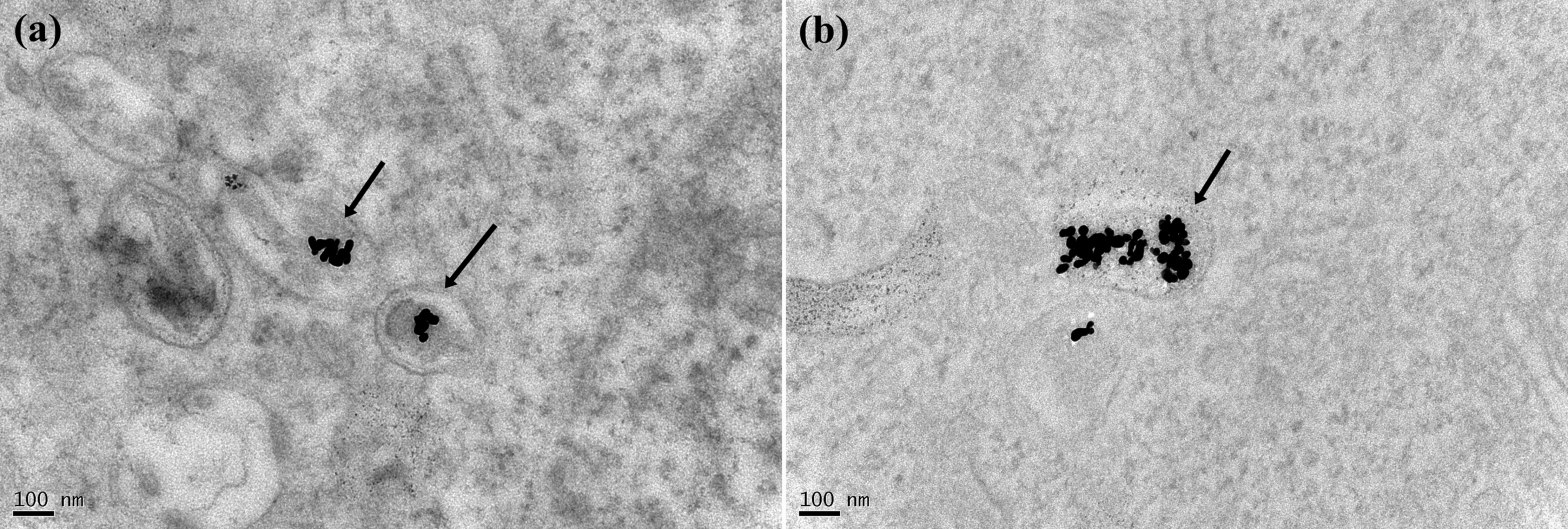
TEM images of MG63 after the uptake of GNPs. TEM images of MG63 cells after 20 hours of treatment with GNPs at concentrations of 1 ppm (a) and 10 ppm (b). GNPs aggregated within the cytoplamic vesicles shown with arrows.

**Figure 2:**
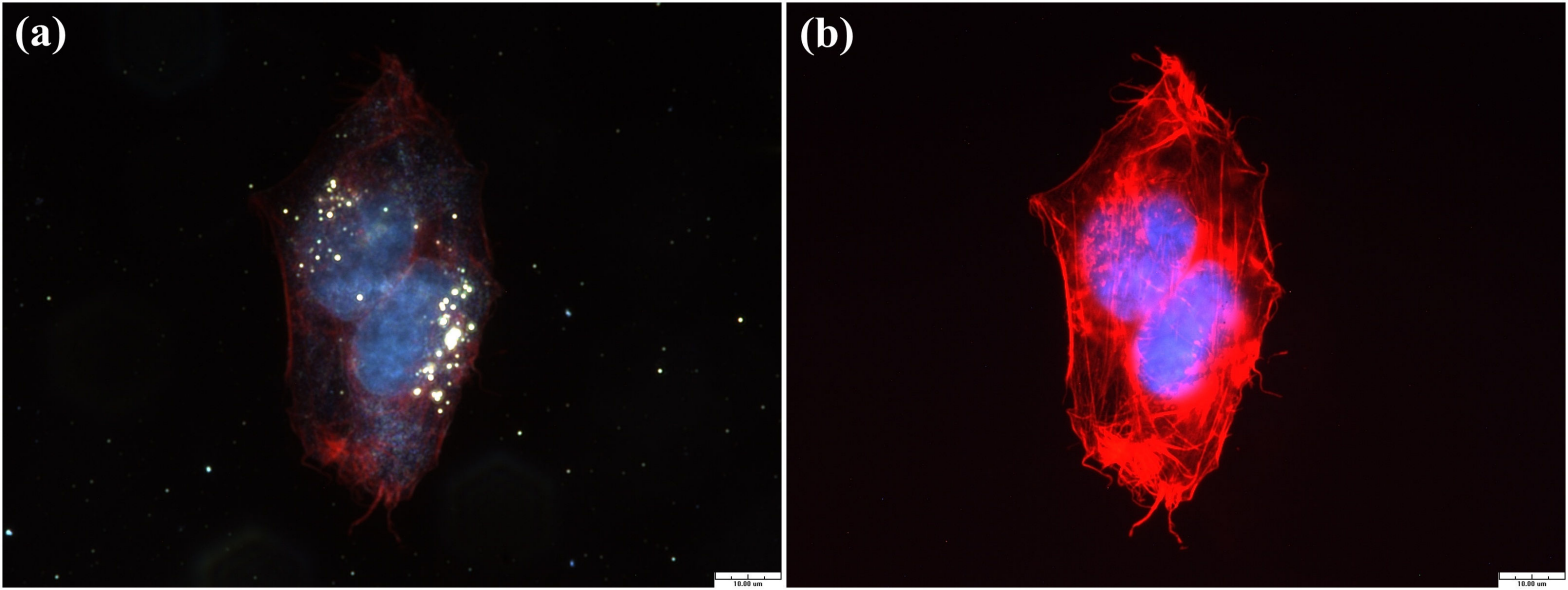
Dark-field images of MG63 after uptake of GNPs. Dark-field (a) scattering and (b) fluorescence images of two MG63 cells undergoing mitosis. Cytoskeletal F-actin (red) are stained with Texas Red-X phalloidin. Cell nuclei (blue) are stained with Hoechst 33258. The bright spots represent endosomes that have enclosed GNPs. Scale bar: 10 µm.

**Figure 3:**
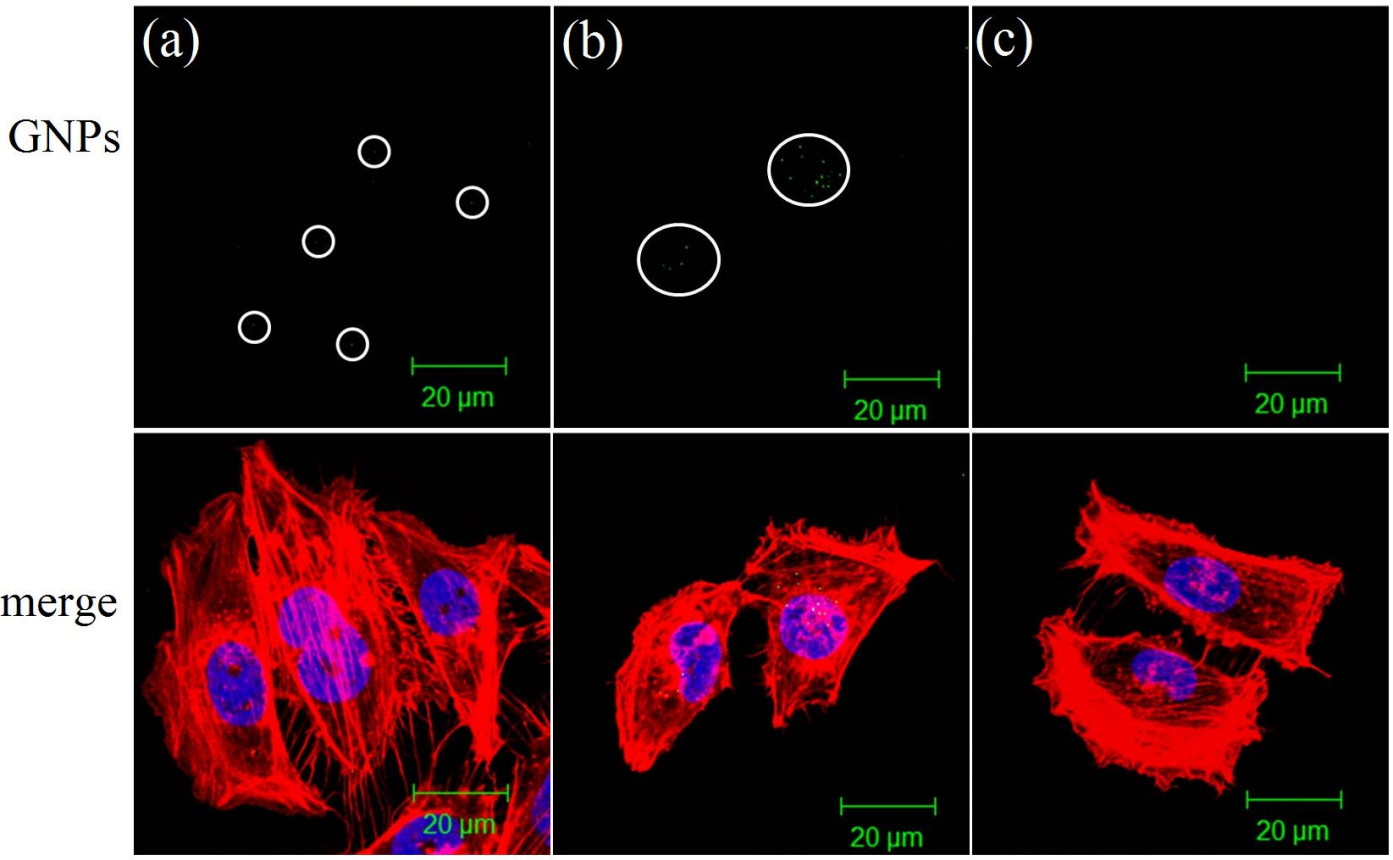
LSCM fluorescence images of MG63 after uptake of GNPs. LSCM fluorescence images of (a) MG63 cells treated with GNPs at a concentration of 1 ppm, (b) 10 ppm, and(c) normally cultured cells. Cytoskeletal F-actin (red) are stained with Texas Red-X phalloidin. Cell nuclei (blue) are stained with Hoechst 33258. The green spots in the circled areas are GNPs.

Figure 1(a) and 1(b) show the TEM images of MG63 treated with GNPs at concentrations of 1 ppm and 10 ppm, respectively, for 20 hours. Both images show that a certain number of GNPs aggregated within the cytoplasmic vesicles, which are the late endosomes. In addition, cells treated with the more concentrated GNP solution took up more GNPs. Subsequently, the nuclei were stained with Hoechst-33258, and the cytoskeleton was stained with Texas Red-X phalloidin to enable the observation of the cell morphology after the uptake of GNPs. Afterwards, we used dark-field microscopy and fluorescence microscopy to simultaneously acquire the scattering and fluorescence images, respectively. The results are shown in Figure 2(a) and (b). In the dark-field image (Figure 2a), the bright spots are the expressions of the endosomes that enclose a certain number of GNPs. In the fluorescence image (Figure 2b), the blue areas are the nuclei and the red areas are the cytoskeleton. In Figure 2, the mitoses of a few of these treated cells are observed; the organelles are separated and move to the opposite sides of the two divided cells. In addition, the LSCM fluorescence images of the treated MG63 show cellular morphologies similar to those of the untreated cells, as shown in Figure 3. Because of the plasmonic light scattering of GNPs, the LSCM scattering images show the endosomes enclosing the aggregated GNPs [33]. Our results indicate that the internalized GNPs do not affect cellular adhesion and spreading.

### Effect of GNPs on Cell Viability, Proliferation, Death Program and Differentiation

**Figure 4:**
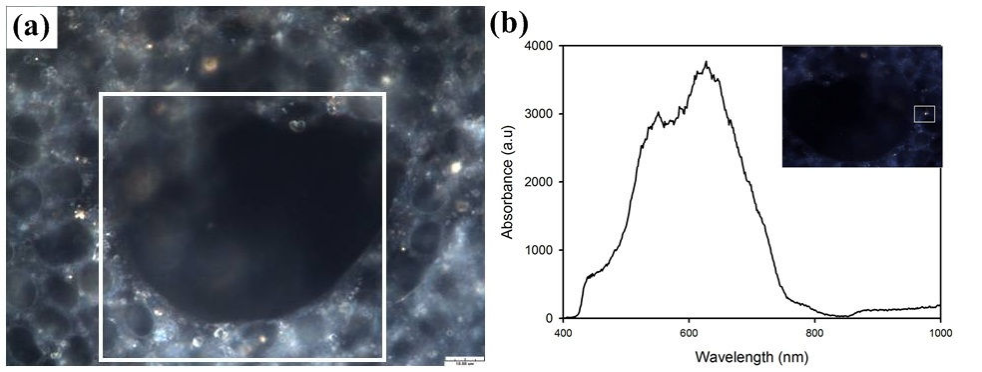
Effect of GNPs on the differentiation of MG63. (a) Dark-field hyperspectral image of MG63 treated with GNPs for 21 days; the image shows the specific nodule-like formation. (b) The corresponding scattering spectrum of the marked endosome.

After MG63 cells were treated with 10 nm GNPs at a concentration of 1 ppm or 10 ppm for 20 hours, the cells were rinsed with phosphate buffer saline (PBS) solution to remove the excess GNPs and then re-cultured in fresh medium without GNPs for 21 days. The formation of nodules in osteoblast cultures indicates containing mineralized precipitates and these precipitates cannot be directly observed under dark-field microscope and LSCM. Therefore, we presumed that darkened areas shown in micrographs were mineralized nodule-like formation. In the dark-field hyperspectral image (Figure 4a), these cells were observed to undergo the differentiation process and to form the specific nodule-like phenotypes [34,35], which is evidence of osteogenetic differentiation and mineralization. Based on the scattering spectrum (Figure 4b) of the endosome, as marked in Figure 4a, we confirmed that the endosomes that enclose GNPs still existed within the cytoplasm for 21 days after a certain number of cellular divisions and differentiations. The spectrum of the light scattered from the endosome indicated that the SPR of the aggregated GNPs was broadened and red-shifted with respect to the light scattered from single GNPs.

**Figure 5:**
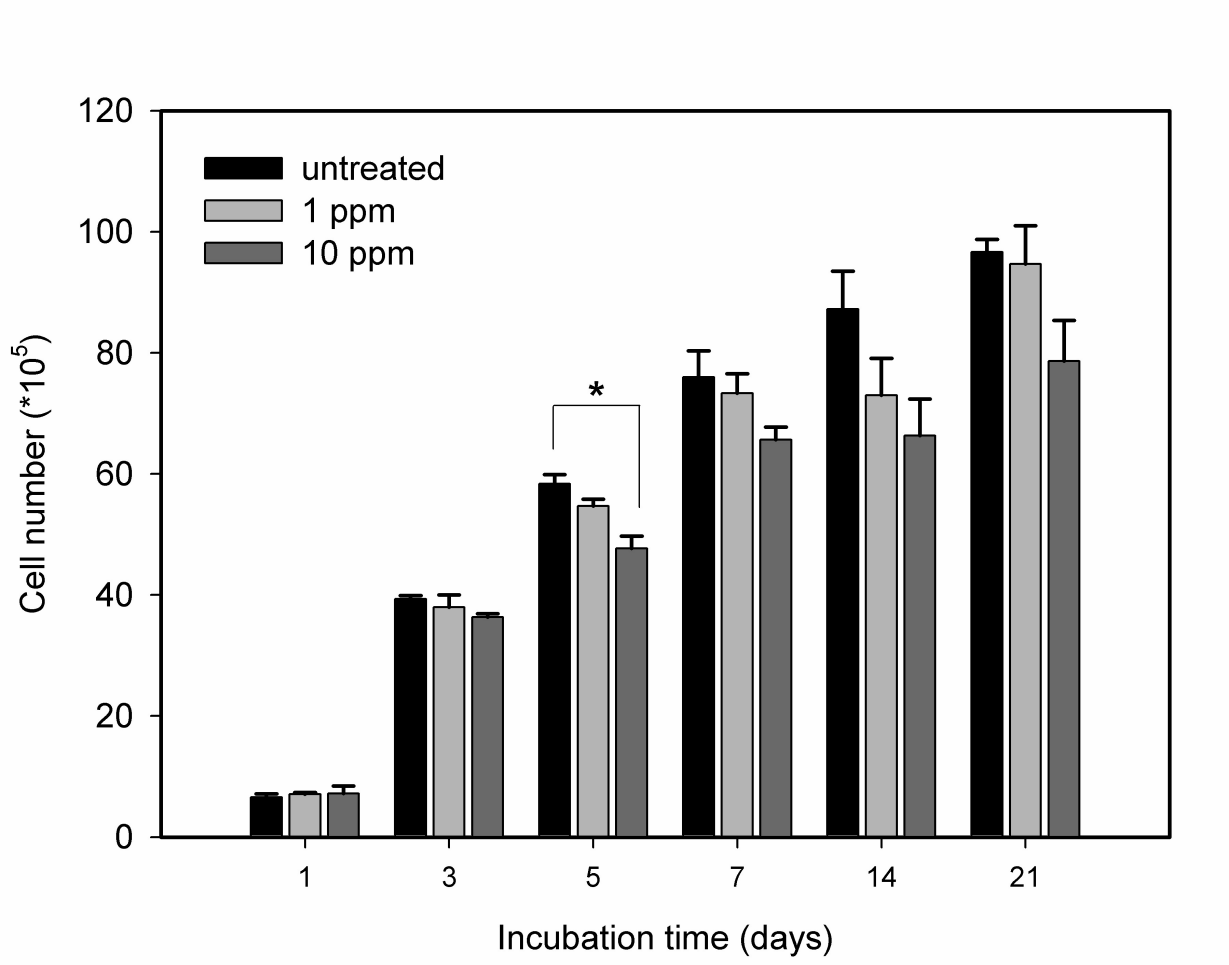
The effect of GNPs on the viability of MG63. The viability of MG63 cells treated with GNPs at a concentration of either 1 ppm or 10 ppm for 20 hours and then cultured in fresh medium for 21 days. Data are presented as the mean ± SD (n=9) and were analyzed using the non-parametric Kruskal-Wallis H-test. Differences at *p* < 0.05 were considered statistically significant.

**Figure 6:**
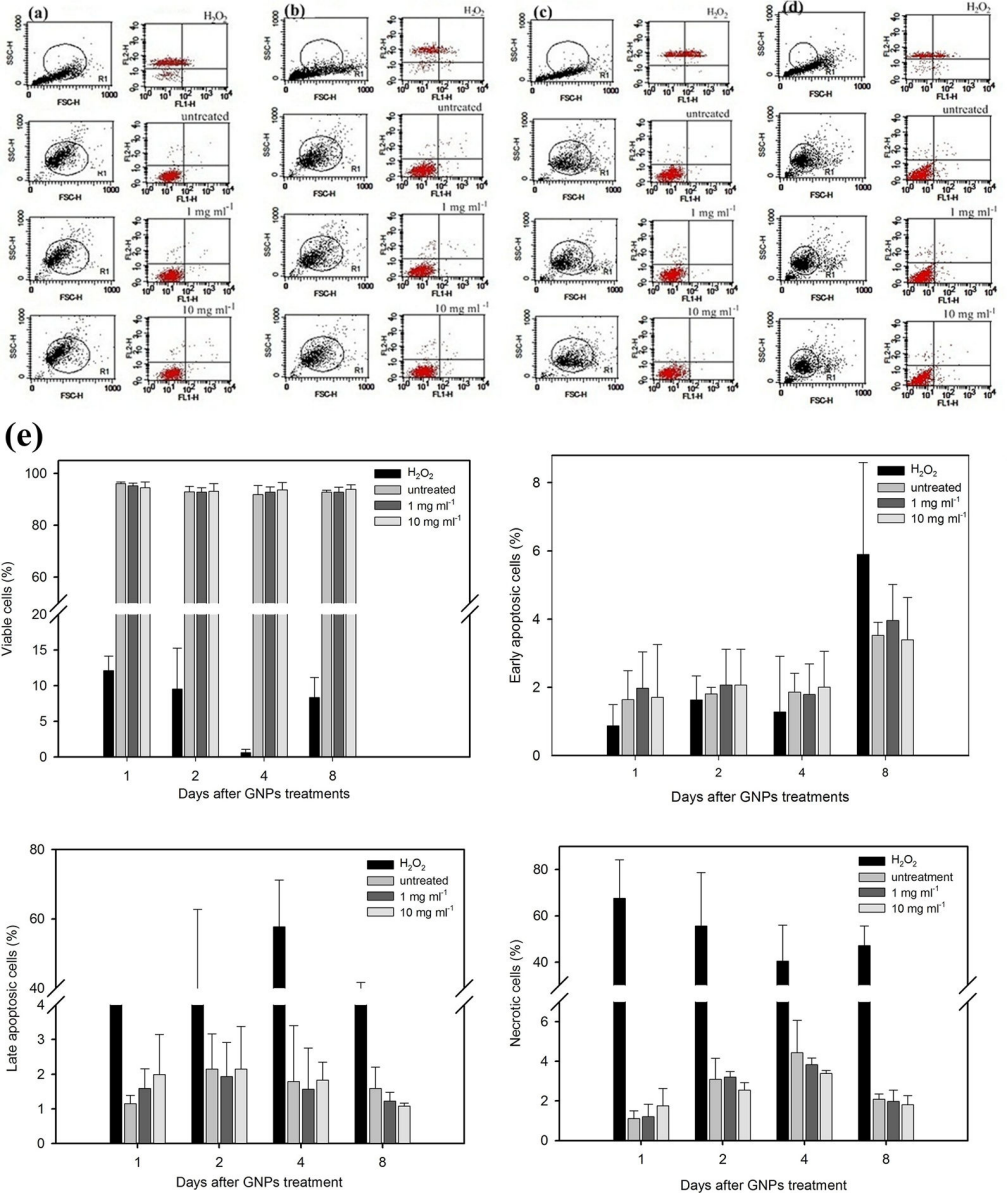
Effect of GNPs on the progressive apoptosis of MG63. The MG63 cells were exposed to H_2_O_2_ (control) and GNPs for 20 hours and then cultured for 1 to 8 days: (a) 1 d, (b) 2 d, (c) 4 d and (d) 8 d. Representative dot plots of Annexin V/PI staining are shown. The upper-left quadrant shows the necrotic (Annexin V-/PI+) population. The upper-right quadrant shows the late apoptotic/necrotic (Annexin V+/PI+) population. The lower-left quadrant shows the vital (Annexin V-/PI-) population. The lower-right quadrant shows the early apoptotic (Annexin V+/PI-) population. The result is from one experiment representative of three similar independent experiments. (e) The percentage of viable cells, early apoptotic cells, late apoptotic cells and necrotic cells after being exposed to GNPs for 20 hours and then cultured for up to 8 days. The results were summarized from three separate experiments and are presented as the mean ± SD. Data were analyzed using the non-parametric Kruskal-Wallis H-test. Differences at *p* < 0.05 were considered statistically significant.

We determined the viability of these cells by counting the number of cells on days 1, 3, 7, 14 and 21. The results are presented in Figure 5. Our results indicate that the growth rates of these treated cells were not significantly affected by the internalized GNPs; all groups exhibited similar growth trends for 21 days (Figure 5; p > 0.05). In addition, the percentages of apoptotic or necrotic cells were measured every two days for the first eight days. We used Annexin V/PI double staining to analyze the effect of GNPs on the cell death program. The results are shown in Figure 6. The four-quadrant plots in each panel are the plots for the necrotic cells (upper left), the late apoptotic cells (upper right), the viable cells (lower left), and the early apoptotic cells (lower right). The cells treated with GNPs revealed four-quadrant plots similar to those of the untreated cells. The percentages of the vital, early apoptotic, late apoptotic and necrotic cells, as quantified using the flow cytometer associated with the Cell Quest software package, are shown in Figure 6(a), (b), (c) and (d). Likewise, no significant difference in the percentages of the early, late apoptosis and necrotic cells was observed between all of the treated and untreated groups. These results illustrate that the internalized GNPs do not alter the cellular apoptosis rate and do not induce necrosis (Figure 6e; p > 0.05). In addition, the cellular attachment and morphology were not altered by GNPs, even though these particles existed inside the cells for more than 21 days.

### Effect of GNPs on MG63 Cell Expression of Osteogenetic Genes

**Figure 7:**
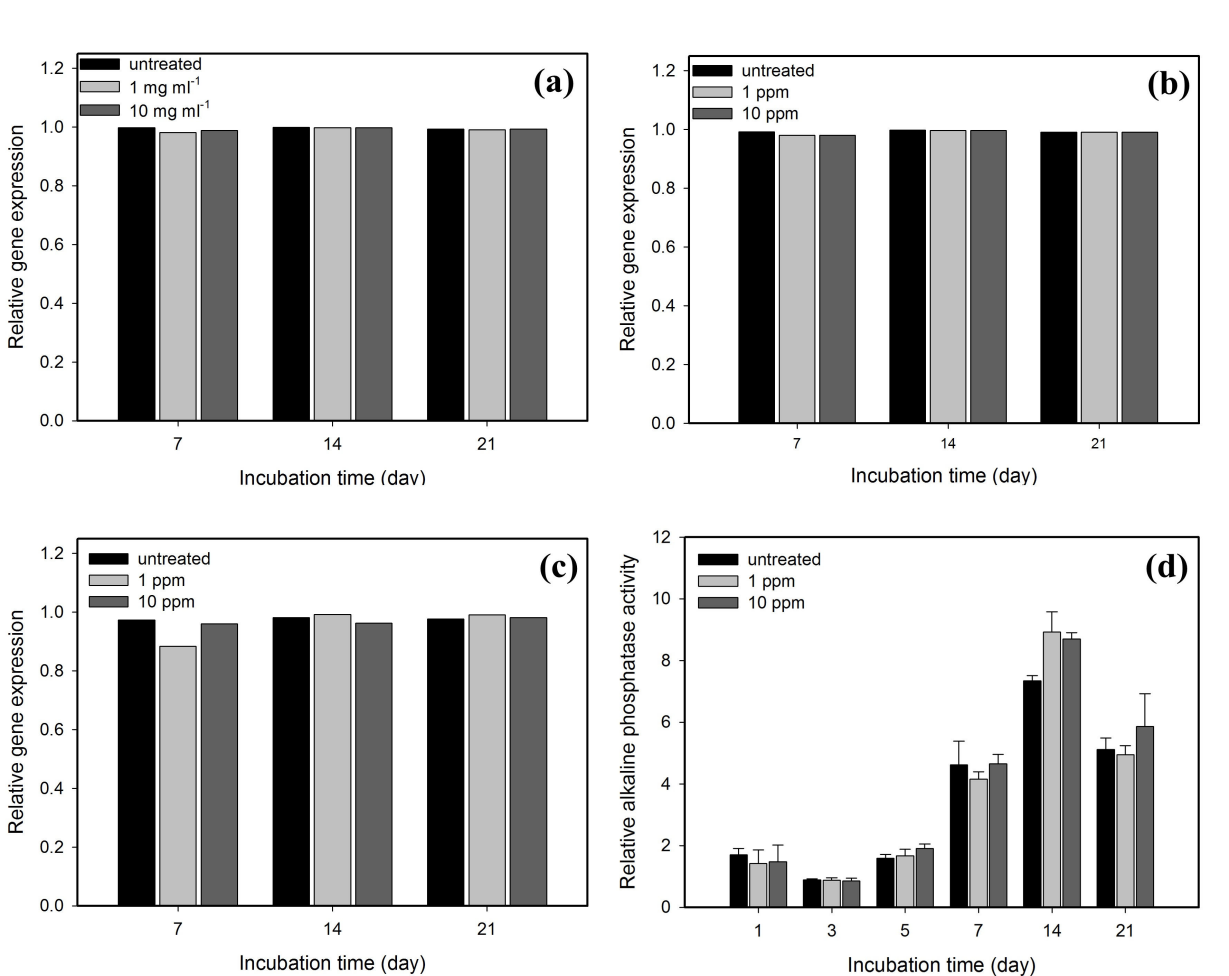
Real-time PCR analyses of the expressions of three bone-associated genes and alkaline phosphatase activity of MG63 treated with GNPs. (a) OPN levels, (b) type I collagen levels, and (c) OCN levels. The gene expression levels are normalized against the 18S ribosomal RNA levels. (d) The alkaline phosphatase activity of the cells. Data are presented as the mean ± SD (n=3). Based on statistical analyses, the expression levels of the three specific bone-associated genes and the alkaline phosphatase activity of the MG63 treated with GNPs show no significant difference in comparison with the control group. Data were analyzed using the non-parametric Kruskal-Wallis H-test. Differences at *p* < 0.05 were considered statistically significant.

The expression levels of genes were analyzed using Q-PCR. Three specific bone-associated gene expression (OPN, type I collagen and OCN) levels of the MG63 osteoblast-like cells treated with GNPs were analyzed using Q-PCR and were normalized against 18S ribosomal RNA levels. These cells were analyzed on days 7, 14 and 21, and the results are shown in Figure 7(a), (b) and (c), respectively. In addition, the phenotypic expression of these cells was evaluated based on measurements of the alkaline phosphatase (ALP) activity after the cells were cultured for up to day 21, as shown in Figure 7d. Our results illustrate that there is no significant difference in the levels of OPN, type I collagen, and OCN gene expression and ALP activity between cells treated with 1 and 10 ppm GNPs and the control group.

## Discussion

The biosafety and biocompatibility of any new biomarker or biomaterial are vital concerns that should be addressed before such materials are applied to biological systems. Table 1 summarizes the previous research on the cytotoxicity of GNPs. Connor et al. found that a variety of surface chemical elements can affect the cell growth [36]. To improve the biocompatibility, Cho et al. conjugated GNPs with polyethylene glycol (PEG) [37]. However, the PEG-modified GNPs induced inflammation and apoptosis of mouse Kupffer cells and macrophages. Moreover, the toxicity of the surfactant CTAB was also pronounced [16]. Therefore, to minimize others chemical effects on the evaluation of the toxicity of GNPs, we fabricated bare GNPs using a simple reduction reaction of gold salt with negative charges as the study target of this work. For TEM-EDS, the copper grid was coated with a carbon film as a support, so copper, carbon and gold are shown in the TEM-EDS spectrum (File S1). However, The EDX spectrum demonstrated that gold was the only chemical element in the nanoparticles. In addition to surface chemical elements, surface charge also plays an important role in the internalization and toxicity of GNPs. Goodman et al. reported that positively charged GNPs had a stronger harmful effect on the viability of cells than negatively charged ones [38]. For our experiment, only bare GNPs with negative Zeta potentials were used (-42.1 ± 0.65 mV). We did not modify these GNPs with any surface layer.

In addition to charge, both size and concentration are key factors that affect the toxicity of GNPs. Shukla et al. [39] and Yen et al. [40] indicated that cell growth was not affected by the concentration of GNPs, but Taylor et al. showed that GNP concentrations greater than 50 µM would inhibit cell growth [41]. In addition, Yi et al. even reported that low concentration of GNPs could promote mesenchymal stem cell osteogenic differentiation [42]. In contrast, Paino et al. reported that GNPs exhibited in vitro genotoxicity and cytotoxicity for cells at very low concentrations [43]. Based on these previous reports, we used 1 ppm and 10 ppm GNPs as low and high concentrations, respectively, to study the concentration effects on long-term co-culture with MG63 cells. Our results showed that the cellular behaviors of MG63 cells were not affected by GNPs regardless of the concentration. By studying size effects, Pan et al. found that the IC50 (half-inhibitory concentration) of GNPs depends on the size of the GNPs but not on the cell type [15]. Chen et al. concluded that GNPs of different sizes had no significant effect over the diameter range from 8 to 37 nm [44]. Based on previous results implies that the cytotoxicity of smaller GNPs is more severe than that of larger GNPs.

In general, small particles are easily taken up by cells via nonspecific endocytosis, and they then leave the cells via exocytosis if the particles cannot be digested by lysosomes. The TEM image in Figure 1 shows that for the cells treated even with a lower concentration of GNPs (1 ppm), the internalized GNPs were enclosed in the vesicles within the cytoplasm rather than being transported to the nucleus or being removed via exocytosis. In principle, GNPs cannot be catabolized by organisms, and the MG63 cell is one type of ECM-producing cell that does not metabolize foreign materials. Therefore, the internalized GNPs should be expelled by MG63 via exocytosis. If the monitoring system (e.g., in vitro microscope) cannot accommodate continuous and long-term culturing, then the whole process (endocytosis and exocytosis) of GNPs in these cells is difficult to observe. Furthermore, if the aggregated GNPs escape from the endosomes, these GNPs would not separate into individual particles because they would be covered with lysosomal enzymes. Thus, we propose that some of the lysosomal enzymes should conjugate onto the surfaces to prevent the re-dispersion of the GNPs. The red-shift in the SPR band of these enclosed GNPs in the late endosomes, as measured using the dark-field hyperspectral system, further confirms the aggregation of GNPs.

In the present study, the cells were treated with a medium containing GNPs for only one day, and they were then washed and incubated with a fresh medium that did not contain GNPs for the following culture days. We found that the GNPs could not be degraded and that they existed in the cytoplasm without inducing any impairing effects for up to 21 days. Thus, we inferred that GNPs were enclosed in late endosomes rather than in early endosome. Because these GNPs always exist in an aggregated form rather than as single particles dispersed within the cytoplasm, the enlarged size prevents the GNPs from entering into the nucleus. In addition, the dark-field images revealed that GNPs would be redistributed into daughter cells during cytokinesis. Finally, the distribution of GNPs within cells decreased with sub-culturing after several cell divisions. These findings are consistent with previous research into the effects of nanodiamond on cellular behaviors [45]. If nanoparticles are taken into cells via non-specific endocytosis, then endocytic vesicles are generated to enclose these nanoparticles. Therefore, larger vesicles reduce the possibility of nanoparticles penetrating into the nucleus or chromosomes. Although GNPs are not biodegradable, they can remain within the endosomes in the cytoplasm for a prolonged period according to our observations. In summary, it is difficult to ensure the biosafety of GNPs with respect to cellular metabolism based on these previous studies. The uncertain cytotoxicity limits the use of GNPs, although their plasmonic optical properties are useful for biomedical applications. We measured the uptake dosage per cell treated with 1 ppm and 10 ppm GNPs for 24, 48 and 72 hours using ICP-OES (File S3). The preliminary data showed that, within 72 hours, the proliferation of the treated MG63 was the same for both concentrations and that the dosage of GNPs per cell decreased as the culture time increased.

In summary, all of our results indicate that bare GNPs with a diameter of 10 nm do not affect cellular behavior if the incubation time is less than the cell doubling time; this result is still true even for a higher concentration of GNPs (10 ppm). We also showed that the location and distribution of GNPs can be monitored using LSCM and dark-field microscopy. However, a quantitative analysis of internalized GNPs using LSCM is worth investigating further [46]. Because the mechanisms of the metabolism of GNPs are still unclear, further investigations are needed for future *in vivo* application of GNP biological systems. Nevertheless, GNPs can be modified with specific ligands to serve as specific cell markers for in vitro optical probing.

## Conclusions

The long-term toxicity effect of GNPs on the division, proliferation and differentiation of a progenitor cell line, MG63 osteoblast-like cells, was studied. These cells were treated with 10 nm GNPs that had a negative surface charge in media that contained 1 ppm or 10 ppm GNPs for more than 20 hours. The TEM, LSCM and dark-field hyperspectral images indicated that the late endosomes in the cells contained aggregated GNPs caused by vesicle fusions. The viability, specific nodule-like phenotypes and gene expression of the treated MG63 after 21 days were almost the same as those of the control group. For the cell death program analysis, the apoptosis and necrosis percentages of the treated cells within 8 days were not significantly different from those of the untreated ones. In addition, the morphology, adhesion and proliferation of GNP-treated MG63 were not affected by the internalized GNPs. In summary, our preliminary results showed low long-term toxicity of GNPs on the osteogenetic differentiation of MG63. In contrast to the toxicity of QDs, the low toxicity and non-biodegradability of GNPs make them a promising biomarker for the long-term optical observation of the differentiation of progenitor or stem cells because of the plasmonic light-scattering of GNPs [25,47]. Moreover, GNPs could also be used for diagnostic and therapeutic applications in clinics, e.g., photoacoustic imaging [48] and photothermal therapy [8,9].

## Supporting Information

File S1 **Energy dispersive spectrum of GNP.** The cupper grid coated with carbon film was used as the supporting grid.
(TIF)

File S2 **The doubling times of treated MG63 by 10 nm GNPs at 1 ppm and 10 ppm and the untreated MG63.** The cells (5 × 10^4^ cells well^-1^) were seeded in 6-well culturing plates and grown to confluence. The cells were then treated with the GNPs at a concentration of either 1 ppm or 10 ppm for an additional 24, 48, 72 or 96 h. A normal culture was used as the control group. The cells were lifted off the culture plate using trypsin and then treated and washed; the cells were subsequently stained with trypan blue and counted with a hemocytometer.
(DOCX)

File S3 **The GNP dosages per cell measured by ICP-OES for the treatment of 1 ppm and 10 ppm GNPs for 24, 48 and 72 h. S5(a) shows the total amount of GNPs taken up by MG63 cells, S5(b) the cell number, and S5(c) the average uptake GNP number per cell.** The cells (5 × 10^4^ cells well^-1^) were seeded in 6-well culturing plates and grown to confluence. The cells were then treated with the GNPs at a concentration of either 1 ppm or 10 ppm for an additional 24, 48, or 72 h. A normal culture was used as the control group. Data were analyzed using the non-parametric Mann–Whitney U-test. Differences at *p* < 0.05 were considered statistically significant.
(TIF)
