# Supplementary figures and images for: Internalized Gold Nanoparticles Do Not Affect the Osteogenesis and Apoptosis of MG63 Osteoblast-Like Cells: A Quantitative, In Vitro Study
Source: PLoS One. 2013 Oct 2;8(10):e76545. doi: 10.1371/journal.pone.0076545 (PMC3788727; doi:10.1371/journal.pone.0076545)

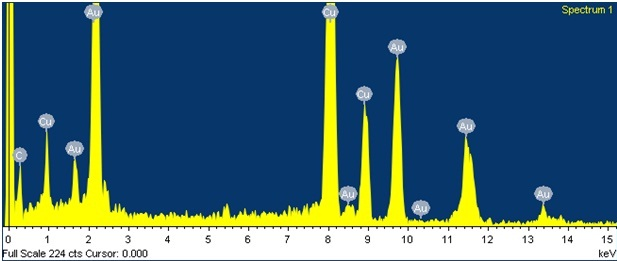

Supplement: File S1 — Energy dispersive spectrum of GNP. The cupper grid coated with carbon film was used as the supporting grid. (TIF) [file pone.0076545.s001.tif]

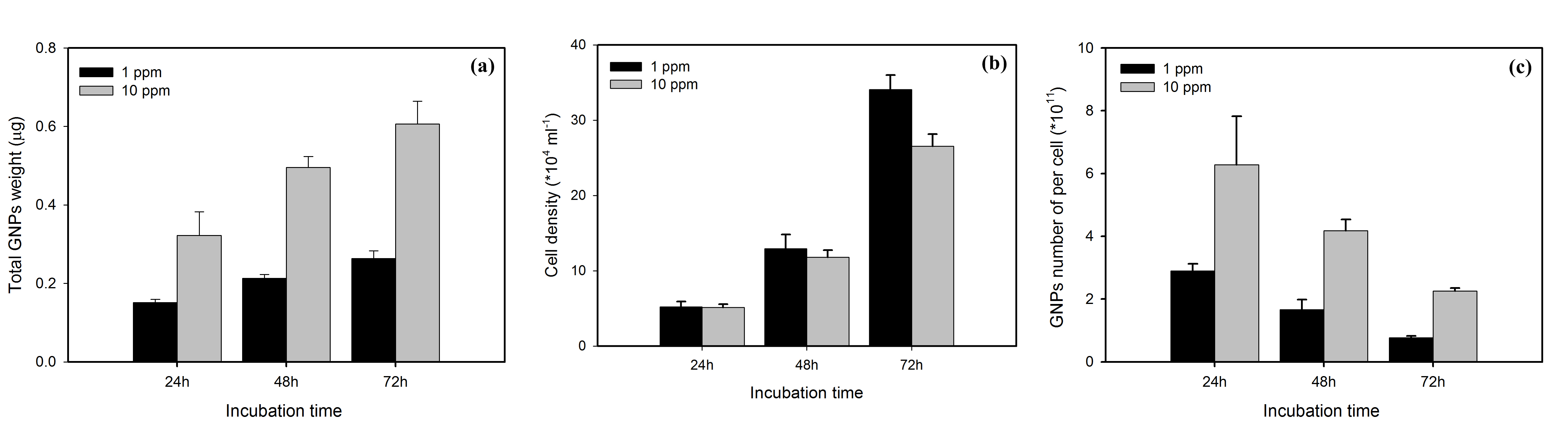

Supplement: File S3 — The GNP dosages per cell measured by ICP-OES for the treatment of 1 ppm and 10 ppm GNPs for 24, 48 and 72 h. S5(a) shows the total amount of GNPs taken up by MG63 cells, S5(b) the cell number, and S5(c) the average uptake GNP number per cell. The cells (5 × 10^4^ cells well-1) were seeded in 6-well culturing plates and grown to confluence. The cells were then treated with the GNPs at a concentration of either 1 ppm or 10 ppm for an additional 24, 48, or 72 h. A normal culture was used as the control group. Data were analyzed using the non-parametric Mann–Whitney U-test. Differences at p < 0.05 were considered statistically significant. (TIF) [file pone.0076545.s003.tif]
