# Supplementary material for: Internalized Gold Nanoparticles Do Not Affect the Osteogenesis and Apoptosis of MG63 Osteoblast-Like Cells: A Quantitative, In Vitro Study
Source: PLoS One. 2013 Oct 2;8(10):e76545. doi: 10.1371/journal.pone.0076545 (PMC3788727; doi:10.1371/journal.pone.0076545)
Supplement: File S2 — The doubling times of treated MG63 by 10 nm GNPs at 1 ppm and 10 ppm and the untreated MG63. The cells (5 × 10^4^ cells well-1) were seeded in 6-well culturing plates and grown to confluence. The cells were then treated with the GNPs at a concentration of either 1 ppm or 10 ppm for an additional 24, 48, 72 or 96 h. A normal culture was used as the control group. The cells were lifted off the culture plate using trypsin and then treated and washed; the cells were subsequently stained with trypan blue and counted with a hemocytometer. (DOCX) [file pone.0076545.s002.docx]

**Supporting Information S2**

|  | untreated | 1 ppm | 10 ppm |
| --- | --- | --- | --- |
| doubling time (hour) | 24.65±1.13 | 24.65±1.13 | 25.17±0.68 |
